# The Diagnostic Accuracy of Urine Lipoarabinomannan Test for Tuberculosis Screening in a South African Correctional Facility

**DOI:** 10.1371/journal.pone.0127956

**Published:** 2015-05-26

**Authors:** Yasmeen Hanifa, Lilanganee Telisinghe, Katherine L. Fielding, Justin L. Malden, Gavin J. Churchyard, Alison D. Grant, Salome Charalambous

**Affiliations:** 1 Aurum Institute, Johannesburg, South Africa; 2 CAPRISA, University of KwaZulu-Natal, Durban, South Africa; 3 London School of Hygiene & Tropical Medicine, London, United Kingdom; 4 School of Public Health, University of Witwatersrand, Johannesburg, South Africa; National Institute for Infectious Diseases (L. Spallanzani), ITALY

## Abstract

**Background:**

We evaluated the diagnostic accuracy of the urine lipoarabinomannan (LAM) antigen detection assay (Clearview TB-ELISA) to screen for tuberculosis in a South African correctional facility.

**Methods:**

Between September 2009 and October 2010, male offenders were screened for tuberculosis (symptoms, chest radiograph, two spot sputum specimens for microscopy and culture), and urine tested for LAM. Sensitivity, specificity and predictive values of LAM were calculated using definite and probable tuberculosis combined as our gold standard.

**Findings:**

33/871 (3.8%) participants (26% HIV-positive) had tuberculosis. Amongst HIV-positive vs. HIV-negative offenders the sensitivity and specificity of LAM was 7.1% vs. 0% and 98.5% vs. 99.8% respectively.

**Conclusion:**

Urine LAM ELISA has inadequate sensitivity for TB screening in this population.

## Introduction

Incarcerated populations worldwide suffer disproportionately high risk of both tuberculosis (TB) [1] and HIV infection,[2] a potent risk factor for TB. In response to the burden of tuberculosis and HIV in its correctional facilities, South Africa recently published national guidelines recommending symptom-based TB screening for all inmates bi-annually, on entry, exit or transfer, and self-presentation, with GeneXpert MTB/RIF as the initial diagnostic test for those requiring further evaluation.[3]

Tests for lipoarabinomannan (LAM), a cell wall lipopolysaccharide specific to mycobacteria that is detectable in urine, are a potentially attractive screening tool for correctional facilities, given the potential for rapid TB diagnosis, the low infection risk posed and ease of sample collection. Evaluations of a commercially available urine LAM ELISA (Clearview TB-ELISA; Alere, USA) have demonstrated sensitivity of 21–38% for culture-proven TB in HIV-positive individuals, [4, 5] which increases to 67% in those with CD4 cell counts <50 cells/μl.[5]

The aim of our study was, within the context of a TB prevalence survey in one of South Africa's largest correctional facilities,[6] to evaluate the diagnostic accuracy of the Clearview TB-ELISA for urine LAM to screen offenders for TB.

## Methods

### Ethics statement

The study was approved by the Research Ethics Committees of the Department of Correctional Services, South Africa, the University of KwaZulu-Natal, South Africa, and the London School of Hygiene & Tropical Medicine, United Kingdom; and the Centre for Disease Control Institutional Review Board. The study was also approved by the Office for Human Research Protections, USA. All participants gave written informed consent, or witnessed verbal consent if unable to write. Consent and participation in the study was voluntary. Participants were able to refuse to take part, with no consequences to their healthcare or any other services as a result of this.

### Study population and procedures

The study site and procedures have been described previously.[6] We enrolled a random sample of offenders who had been incarcerated for at least six months ("currently incarcerated") and a consecutive sample of "newly sentenced" offenders. Individuals with an expected stay of less than 3 months in the study facility were excluded to ensure follow-up of medical records.

All participants underwent a standardised symptom-screening questionnaire (any symptom compatible with tuberculosis, including cough, fever, night sweats, or unintentional weight loss), chest radiography (assessed by two readers using a standardised tool), and provided two spot sputum specimens for smear and mycobacterial culture. Urine for anonymised HIV testing was collected from those consenting, and an additional urine sample stored at -20°C on the day of collection for subsequent LAM measurement. Correctional facility medical records were reviewed 3 months post-enrolment to ascertain any additional TB diagnoses made within three months of enrolment and thus strengthen the gold standard. HIV counselling and testing was provided for offenders who wished to know their HIV status.

Sputum specimens underwent fluorochrome microscopy and liquid culture using the Mycobacterial Growth Indicator Tube (BACTEC-MGIT 960). Positive mycobacterial cultures were speciated using the GenoType Mycobacterium CM kit (Hain Lifescience, Nehren, Germany). Anonymised urine samples were tested for HIV antibodies using the MAXIM HIV-1 urine EIA (Maxim Biomedical Inc, MD, USA). Urine LAM testing was performed on stored samples in batches using Clearview TB ELISA (Inverness Medical Innovations, Scarborough, ME, USA).

### Case definition

TB was classified as *definite* if one sputum culture was positive for *M*. *tuberculosis* and there were either compatible clinical or radiological features, or additional microbiological confirmation (any grade of smear or further positive culture); *probable* if either only one culture was positive without compatible clinical or radiological features; or ≥1 sputum smear-positive (≥grade 1+) and culture-negative; *possible* if classical radiological features on consensus (pleural effusion, cavitation, or upper lobe changes); or ≥1 scanty positive smear and culture-negative, without compatible clinical or radiological features.

### Statistical analysis

We calculated sensitivity, specificity and predictive values of the LAM assay, using definite and probable tuberculosis combined as our gold standard. We excluded possible TB cases, those already on TB treatment at enrolment, and those without a full set of TB screening investigations from this analysis.

## Results

**Fig 1:**
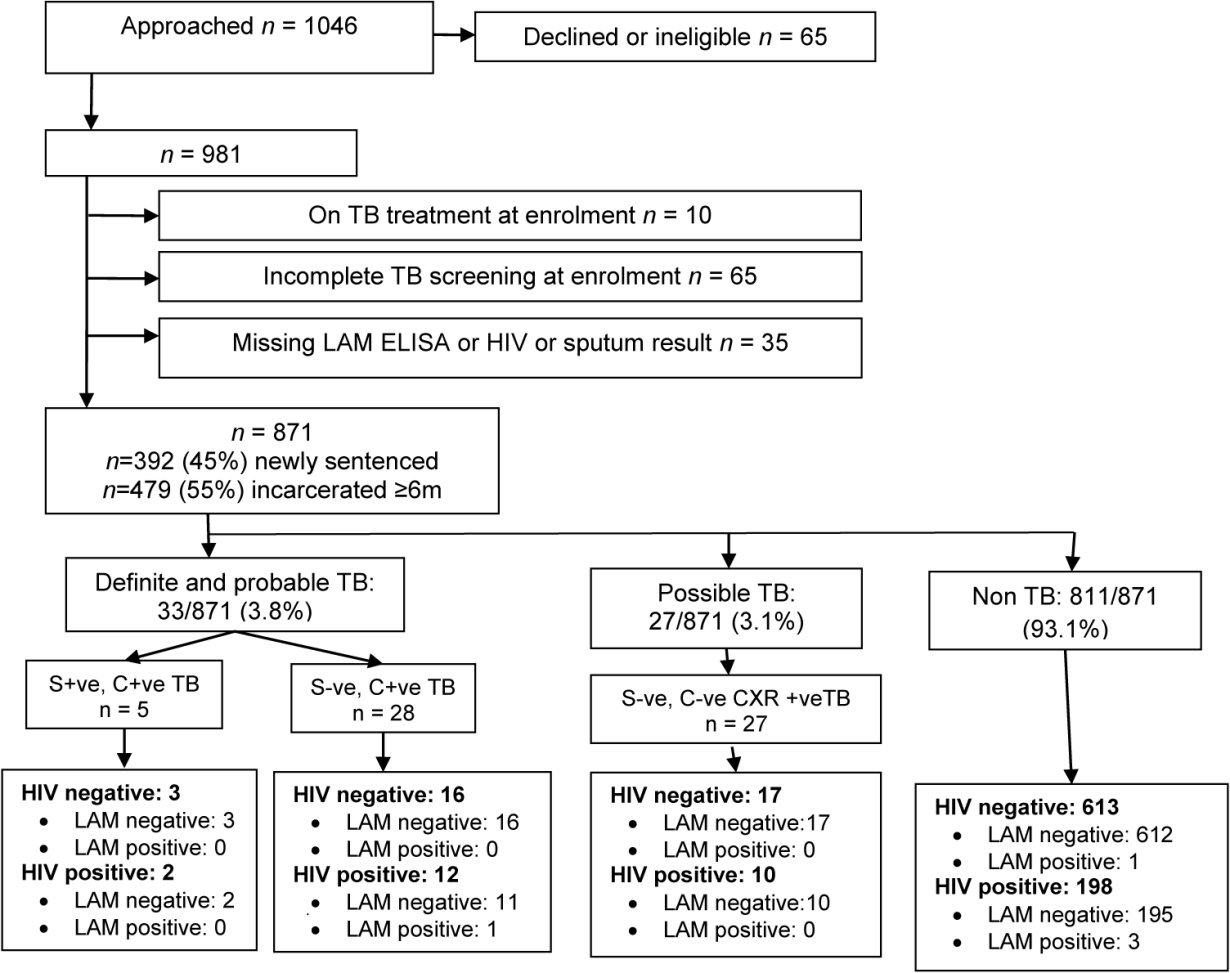
Participant flow chart. LAM = lipoarabinomannan; TB = tuberculosis; S +ve = sputum smear-positive for acid-fast bacilli; S –ve = sputum smear-negative for acid-fast bacilli; C +ve = sputum culture-positive for *M*. *tuberculosis*; C –ve = sputum culture-negative for *M*. *tuberculosis*; CXR +ve = classical radiographic features of TB.

Fig 1 summarises study inclusions, exclusions, case definitions, and urine LAM test results. Between September 2009 and October 2010, a total of 981 offenders were enrolled to the study. Amongst 871 evaluable participants, 871 (100%) were male, 812 (93%) were Black African, the median age was 32 (interquartile range [IQR] 27–38) years, 275 (32%) shared a cell with >50 offenders, and 110 (12.6%) reported at least one previous course of TB treatment. Median incarceration time in “currently incarcerated” offenders was 74 (IQR 43–103) months, and median detention in remand for new entrants was 14 (IQR 6–27) months. 222/871 (25.5%) offenders had a positive urine HIV test result.

33/871 (3.8%) participants fulfilled our case definition for definite and probable TB combined; five were smear-positive and 12 had any symptom compatible with TB. TB prevalence was greater amongst HIV-positive compared with HIV-negative offenders, 6.6% (14/212) vs. 3.0% (19/632), (*P* = 0.02).

5/871 (0.6%) participants had a positive LAM test result, of whom one was HIV-positive and sputum culture-positive for *M*. *tuberculosis*. Table 1 summarises the performance of the urine LAM test in our study population. Amongst HIV-positive offenders the sensitivity and specificity of the LAM assay were 7.1% (95% confidence interval [CI] 0.2%, 33.9%) and 98.5% (95% CI 95.6%, 99.7%) respectively. Amongst HIV-negative offenders the sensitivity and specificity of the LAM assay were 0% and 99.8% (95% CI 99.1%, >99.9%) respectively. The only TB case correctly identified by the urine LAM test was in an HIV-positive individual who was smear-negative, culture-positive, with compatible clinical but no radiological features of TB.

**Table 1:** Performance of urine LAM test overall and stratified by HIV status compared with gold standard of bacteriologically-confirmed TB.

Group	Prevalence of TB	Prevalence of positive LAM	Sensitivity	Specificity	PPV	NPV
	% (n)	% (n)	% (95% CI)	% (95% CI)	% (95% CI)	% (95% CI)
**Overall (n = 844)**	3.9 (33)	0.6 (5)	3.0 (0.1–15.8)	99.5 (98.7–99.9)	20.0 (0.5–71.6)	96.2 (94.7–97.4)
**HIV-positive (n = 212)**	6.6 (14)	1.9 (4)	7.1 (0.2–33.9)	98.5 (95.6–99.7)	25.0 (0.6–80.6)	93.8 (89.5–96.6)
**HIV-negative (n = 632)**	3.0 (19)	0.2 (1)	0 (0–17.6)	99.8 (99.1->99.9)	0 (0–97.5)	97.0 (95.3–98.2)

NPV = negative predictive value; PPV = positive predictive value; CI confidence interval

27 participants with possible TB have been excluded from this analysis

## Discussion

Our study has shown that the sensitivity of Clearview TB-ELISA is too low to be useful for the general screening of offenders in this correctional facility, of whom the majority are HIV-negative. Sensitivity was greater in those who were HIV-positive, in keeping with findings from a recent meta-analysis.[7] Indeed Clearview TB-ELISA is licensed as a screening test only in HIV-positive TB suspects. Evaluations amongst HIV-infected TB suspects have suggested that lateral flow urine test for LAM (Determine TB-LAM; Alere, USA) may be useful to rule-in TB in hospitalised patients with advanced immunosuppression with sensitivity and specificity of 66% for culture-proven TB [8]; and that the sensitivity of combination of smear microscopy with lateral flow LAM (72%) approaches that of Xpert MTB/RIF alone, [9] the latter study suggesting that these tests identified different groups of patients with TB. One limitation of our study is the lack of CD4 cell counts in those offenders confirmed to be HIV positive, and a possible explanation for the very low sensitivity (7%) amongst HIV-positive offenders, compared with other published data may be higher median CD4 cell count in this “healthier” population.

Despite the high prevalence of both HIV and TB among offenders, the low sensitivity of the urine LAM ELISA precludes recommendation of this test as a screening test for TB in this population. The results of our study reinforce the knowledge that the LAM assay, as reported previously in the literature, is not useful in populations with unknown HIV status and CD4 cell counts. [4, 7]

## References

[1] Baussano I, Williams BG, Nunn P, Beggiato M, Fedeli U, Scano F. Tuberculosis incidence in prisons: a systematic review. PLoS Med. 2010;7(12):e1000381 10.1371/journal.pmed.1000381 21203587 PMC3006353

[2] United Nations Office on Drugs and Crime. HIV/AIDS Prevention, Care, Treatment and Support in Prison Settings. A Framework for an Effective National Response Vienna2006 [2011 December 12]. Available: **http://www.unodc.org/pdf/HIV-AIDS_prisons_July06.pdf**.

[3] Department of Health—Republic of South Africa. Guidelines for the management of Tuberculosis, Human Immunodeficiency Virus and Sexually-Transmitted Infections in Correctional Centres 2013 2013 [2014/06/15]. Available: http://www.section27.org.za/wp-content/uploads/2013/05/Guidelines-for-the-management-of-Tuberculosis-Human-Immunodeficiency-Virus-and-Sexually-Transmitted-Infections-in-Correctional-Centres-2013.pdf.

[4] Dheda K, Davids V, Lenders L, Roberts T, Meldau R, Ling D, et al Clinical utility of a commercial LAM-ELISA assay for TB diagnosis in HIV-infected patients using urine and sputum samples. PLoS One. 2010;5(3):e9848 10.1371/journal.pone.0009848 20352098 PMC2844421

[5] Lawn S, Edwardsa D, Kranzer K, Vogta M, Bekker LG, Wood R. Urine lipoarabinomannan assay for tuberculosis screening before antiretroviral therapy diagnostic yield and association with immune reconstitution disease. AIDS. 2009;23(14):1875–80. 20108382 10.1097/qad.0b013e32832e05c8

[6] Telisinghe L, Fielding KL, Malden JL, Hanifa Y, Churchyard GJ, Grant AD, et al High Tuberculosis Prevalence in a South African Prison: The Need for Routine Tuberculosis Screening. PLoS One. 2014;9(1):e87262 10.1371/journal.pone.0087262 24498059 PMC3907552

[7] Minion J, Leung E, Talbot E, Dheda K, Pai M, Menzies D. Diagnosing tuberculosis with urine lipoarabinomannan: systematic review and meta-analysis. Eur Respir J. 2011;38(6):1398–405. 10.1183/09031936.00025711 21700601

[8] Peter JG, Theron G, van Zyl-Smit R, Haripersad A, Mottay L, Kraus S, et al Diagnostic accuracy of a urine lipoarabinomannan strip-test for TB detection in HIV-infected hospitalised patients. Eur Respir J. 2012;40(5):1211–20. 10.1183/09031936.00201711 22362849 PMC5523653

[9] Lawn SD, Kerkhoff AD, Vogt M, Wood R. Diagnostic accuracy of a low-cost, urine antigen, point-of-care screening assay for HIV-associated pulmonary tuberculosis before antiretroviral therapy: a descriptive study. Lancet Infect Dis. 2012;12(3):201–9. 10.1016/S1473-3099(11)70251-1 22015305 PMC3315025

